# The Role of Nozzle Temperature, Bed Temperature, and Post-Treatment Annealing Temperatures in Optimizing Tensile and Flexural Strength of FDM-Printed PEEK

**DOI:** 10.3390/polym18141694

**Published:** 2026-07-09

**Authors:** Sundarakannan Rajendran, Sakthivel Sankaran, Yo-Lun Yang, Kinga Korniejenko, Thirumalai Kumaran Sundaresan, Uthayakumar Marimuthu, Koppiahraj Karuppiah

**Affiliations:** 1 Department of Mechanical Engineering, Vel Tech Rangarajan Dr. Sagunthala R&D Institute of Science and Technology, Avadi, Chennai 600062, Tamil Nadu, India; drsundarakannanr@veltech.edu.in; 2 Department of Biomedical Engineering, Kalasalingam Academy of Research and Education, Krishnankovil 626126, Tamil Nadu, India; sakthivel@klu.ac.in; 3 Graduate Institute of Manufacturing Technology, National Taipei University of Technology, Taipei 10608, Taiwan; y.yang@ntut.edu.tw; 4 Faculty of Materials Engineering and Physics, Cracow University of Technology, 37 Jana Pawła II Str., 31864 Cracow, Poland; kinga.korniejenko@pk.edu.pl; 5 Department of Mechanical Engineering, PSG Institute of Technology and Applied Research, Coimbatore 641062, Tamil Nadu, India; thirumalaikumaran@psgitech.ac.in; 6 Department of Mechanical Engineering, Amrita Vishwa Vidyapeetham, Amritapuri, Kollam 690525, Kerala, India; uthay@am.amrita.edu; 7 Department of Mechanical Engineering, KIT-Kalaignarkarunanidhi Institute of Technology, Coimbatore 641402, Tamil Nadu, India

**Keywords:** fused deposition modelling, PEEK, mechanical properties, response surface methodology

## Abstract

Fused deposition modelling (FDM) is increasingly used to produce high-performance polymer components; however, the mechanical performance of printed parts is often limited by weak interlayer adhesion, void formation, and residual thermal stresses. In this study, the effects of nozzle temperature, bed temperature, and post-treatment annealing temperature on the tensile and flexural strength of FDM-printed polyether ether ketone (PEEK) were investigated and optimized using Response Surface Methodology (RSM). A face-centred central composite design was employed to evaluate the individual, quadratic, and interaction effects of the three thermal parameters. The results showed that post-treatment annealing temperature was the most influential factor, contributing 56.48% to tensile strength and 52.73% to flexural strength, followed by nozzle temperature, which contributed 30.56% and 30.15%, respectively. Bed temperature showed a comparatively smaller individual effect; however, its interaction with nozzle temperature significantly influenced both tensile and flexural strength. The confirmation experiment performed at 200 °C post-treatment temperature, 414 °C nozzle temperature, and 142 °C bed temperature produced a tensile strength of 55.65 MPa and a flexural strength of 81.08 MPa, with prediction errors of 5.63% and 4.08%, respectively. SEM fracture analysis provided qualitative evidence that improved thermal processing reduced interlayer separation and visible void-related defects while promoting a more cohesive fracture morphology. These improvements are attributed to enhanced interlayer fusion, possible polymer-chain diffusion across layer boundaries, and thermal-stress relaxation during annealing. The findings demonstrate that thermal-parameter optimization and post-treatment annealing can improve the mechanical performance of FDM-printed PEEK within the investigated processing window.

## 1. Introduction

Fused deposition modelling (FDM) is one of the most widely used additive manufacturing methods because it is inexpensive, flexible, and able to process a range of materials. However, although FDM-printed parts are increasingly used in industry, mechanical drawbacks frequently become apparent in FDM-printed parts, especially because of the layer-by-layer deposition technique [1,2]. This can cause poor interlayer bonding, which lowers the tensile and flexural strength and limits the use of FDM parts in high-performance applications [3]. Therefore, improving the mechanical properties of FDM-printed parts remains an important research area. The process parameters that are sensitive to temperature such as the nozzle temperature, bed temperature and the post-treatment annealing temperature are important factors that determine the mechanical behaviour of FDM parts [4]. The extrusion of the filament is regulated by the nozzle temperature in order to have the appropriate flow of material and to obtain adhesion between layers [5,6]. It is the bed temperature that keeps the lower layers at the correct temperature preventing warping and enhancing the overall stability of the part [7,8]. Post-treatment annealing can improve printed interfaces by promoting stress relaxation and interlayer bonding [9,10]. The aim of the study is to conduct a systematic study on the influence of these three temperature parameters on tensile and flexural strength of FDM parts with Response Surface Methodology (RSM) and central composite design (CCD).

Optimization of the process parameters to create mechanically strong components has been a major concern in the study of FDM-printed polymeric material [11]. Different researchers have also examined various factors to enhance the mechanical properties of FDM-printed polymers [12]. In particular, recent studies have applied formal design-of-experiments approaches, especially Response Surface Methodology based on central composite designs with factorial, axial, and centre points, together with ANOVA-based factor screening and model-validation procedures, to optimize the mechanical performance of 3D-printed polymer and polymer-composite systems [13,14]. For example, Foppiano et al. [15] investigated the improvement of tensile properties in Fused Filament Fabrication (FFF)-printed acrylonitrile butadiene styrene (ABS) and reported that a nozzle temperature of 231 °C produced the maximum tensile strength. Similarly, Shuto et al. [16] studied the influence of nozzle temperature on the interlaminar tensile strength of carbon fibre-reinforced polyphenylene sulfide (CF/PPS) composites. Their results showed that the highest strength was obtained within the nozzle temperature range of 310–330 °C, while a lower strength was observed at 320 °C. This variation was attributed to changes in crystallinity and void content. Although higher crystallinity may reduce toughness, it was not strongly correlated with strength, indicating that further optimization of the printing process is required.

Aliheidari et al. [17] in a separate study examined the influence of bed temperature on the mechanical performance of ABS that was printed using FDM by a fracture mechanics methodology. The temperature of the bed was discovered to enhance the bonding of the printed material with the build plate, minimizing the problem of warping or layer lifting, particularly when using materials with a tendency to shrink on cooling such as ABS. Furthermore, keeping the bed temperature elevated served to keep the layers in contact with their glass transition temperature (Tg), which improved adhesiveness between them. In spite of the usual positive effect of bed temperature on fracture resistance, enhancing stronger bonding, it was not as strong as that of nozzle temperature, which was more important in material flow and fusing the layers [18]. It is notable that the correlation between interlayer adhesion and bed temperature was non-linear and that higher bed temperature past a specific limit did not increase adhesion with already fused layers. Choi et al. [19] also supported the relevance of bed temperature and stated that low bed temperature did not result in adequate interlayer adhesion, whereas high temperatures resulted in optimal interlayer adhesion and reduced heat-shrinkage defects. The higher bed temperatures enabled the material to solidify more slowly and therefore the material hardened over a longer duration, thereby decreasing thermal stresses and enhancing dimensional precision.

The effect of nozzle temperature on the mechanical properties of 3D-printed PLA composites in Elhattab et al. [20] was found to increase as the nozzle temperature was raised from 190 to 220 °C, which improved the tensile strength and stiffness. This was attributed to the greater mobility and alignment of the molecules at higher temperature, so the material was considered suitable for load-bearing use. On the same note, the study by Alsoufi et al. [21] also established that the lower the nozzle temperatures, the smaller the warping deformation of FDM-printed components, as it enhanced the adhesion of layers and minimized the thermal loads which normally lead to warping. This is what their results indicated; the higher the nozzle temperature, the lower the dimensional errors and warping, which is crucial when it comes to manufacturing quality functional components [22]. Although these studies have been able to discuss the effects of nozzle and bed temperature as independent variables, the combined effect of these variables, as well as post-treatment annealing, has not been fully reported. More studying of the joint actions of such temperature-related FDM printing factors is required in order to realize the full picture and efficiency of FDM-printed materials. Moreover, most of this work concerns lower-performance thermoplastics such as ABS and PLA. For PEEK, a high-performance semi-crystalline polymer whose strength is strongly governed by crystallinity and interlayer fusion, the combined and interacting influence of nozzle temperature, bed temperature, and post-treatment annealing temperature has not been quantified within a single statistically designed experiment. The present study addresses this gap by using RSM and a face-centred CCD to model the individual, quadratic, and interaction effects of these three thermal parameters on the tensile and flexural strength of FDM-printed PEEK, and to identify the parameter combination that maximizes mechanical performance on a standard desktop-class printer.

In this study, the tensile and flexural strengths of FDM-printed PEEK were investigated using Response Surface Methodology (RSM), with three independent parameters, namely, nozzle temperature, bed temperature, and post-treatment temperature. The mechanical properties were then statistically analyzed with regard to the effect of these factors. This study might allow a more in-depth insight into the joint action of these temperature-related parameters on the work of FDM-printed PEEK, which might help understand the necessary process parameters to achieve maximum strength and durability. Moreover, the obtained results can be used to create more stable and high-performing FDM-printed components that can be used in the industrial environment with intensive loads.

## 2. Materials and Methods

### 2.1. FDM Printing and Testing

PEEK was used to fabricate tensile and flexural test specimens by FDM on a CreatBot PEEK-250 high-temperature 3D printer, which provides a nozzle temperature of up to 480 °C, a platform temperature of up to 200 °C, and an actively heated, fully enclosed chamber that can reach 200 °C. PEEK filament (product code PEEK K10; Kexcelled, Suzhou, China) of 1.75 mm diameter was used. The supplier reports a density of 1.28 g/cm^3^ and an elongation at break of 4–6% for this material; the data sheet additionally lists a tensile strength of 70–80 MPa, which corresponds to printed PEEK K10 specimens tested as per ISO 527 (International Organization for Standardization, ISO 527-1:2019 Plastics—Determination of tensile properties—Part 1: General principles, 3rd ed. Geneva, Switzerland: ISO, 2019.) under the supplier’s optimized conditions rather than to the as-received filament. All specimens were printed with a 0.4 mm nozzle at a layer height of 0.2 mm, an infill density of 80%, a 0° build orientation, and a print speed of 20 mm/s, with the heated chamber maintained at 120 °C. The nozzle temperature (380, 400, and 420 °C) and the bed temperature (120, 140, and 160 °C) were varied to study their effects, while all other parameters were held constant. After printing, the specimens were annealed for 1 h at 100, 150, or 200 °C and then cooled to room temperature. Prior to printing, the PEEK filament was dried at 120 °C for 4 h in a hot-air oven and stored in a sealed desiccator until use, in order to remove absorbed moisture and to avoid moisture-related defects such as bubble formation, unstable extrusion, and interlayer voids. The bed-temperature range of 120–160 °C was selected on the basis of the stable operating window of the printer and the need to avoid excessive thermal deformation and warping during specimen fabrication; although PEEK has a glass transition temperature close to this range and higher bed temperatures could further reduce thermal gradients and improve interlayer bonding, the upper limit was kept at 160 °C to maintain stable, distortion-free printing. The post-treatment annealing range of 100–200 °C was likewise chosen to bracket the glass transition temperature of PEEK (~143 °C): the upper levels (150–200 °C) provide sufficient chain mobility for residual-stress relaxation, annealing, and interlayer diffusion, while the whole range remains well below the melting region (~343 °C) so that the as-printed specimen geometry is preserved without melting, slumping, or distortion. Annealing closer to the crystallization/melting region, although potentially increasing crystallinity [23], carries a substantial risk of geometric deformation of the free-standing printed specimens. The build (layer) orientation and the infill density were deliberately held constant so that the effects of the three thermal parameters (post-treatment, nozzle, and bed temperature) could be isolated; although these geometric factors are known to influence the mechanical behaviour of FDM parts, including them would have substantially increased the number of experimental runs required by the RSM design, and they were therefore considered beyond the present scope and reserved for future work. For the same reason, the chamber temperature was held constant at 120 °C throughout all experiments and was not included among the optimized factors of the experimental design. Although the chamber temperature strongly influences interlayer bonding and warping in PEEK, treating it as a fourth factor would have substantially increased the number of runs required by the RSM design; it was therefore fixed as a controlled condition in this first study to isolate the effects of the three thermal parameters, and its optimization is reserved for future work.

Flexural tests were performed in accordance with ASTM D790 (: ASTM International, ASTM D790: Standard Test Methods for Flexural Properties of Unreinforced and Reinforced Plas-tics and Electrical Insulating Materials. West Conshohocken, PA, USA: ASTM International.) using three-point bending on specimens measuring 125 mm × 12.7 mm × 3.2 mm, with a support span of 51.2 mm (16:1 span-to-depth ratio) at a crosshead speed of 2 mm/min. Tensile tests were performed in accordance with ASTM D638 ( ASTM International, ASTM D638: Standard Test Method for Tensile Properties of Plastics. West Conshohocken, PA, USA: ASTM International.) on Type I specimens (gauge length 50 mm, width 13 mm, thickness 3.2 mm, total length 165 mm) at a crosshead speed of 2 mm/min. Both tests were conducted on a universal testing machine following the ASTM D638 and ASTM D790 procedures [24]. Three specimens were tested per run, and all reported values represent the mean ± standard deviation. This triplicate testing is consistent with common practice in FDM and RSM parameter studies; moreover, the face-centred design includes four replicated centre points, which provide an independent estimate of pure experimental error and enable the lack-of-fit test reported in the ANOVA, so that repeatability is captured beyond the per-run triplicates. The fracture surfaces were examined by scanning electron microscopy (SEM) to identify the microstructural failure mechanisms.

### 2.2. Experimental Design with RSM

The experimental design in this study was developed using the central composite design (CCD) method in Design-Expert v12 software. CCD is a subset of Response Surface Methodology (RSM), a statistical approach widely used to investigate the relationship between input factors and output responses in a systematic and organized manner [25,26]. In this work, RSM was employed both to characterize the influence of the selected printing parameters on the tensile and flexural strength of FDM-printed PEEK samples and to identify the optimum combination of input parameters for maximizing these properties.

A three-factor CCD was used. The design combines three types of points: factorial points, axial (star) points, and centre points. The two-level factorial portion, coded −1 and +1 (the lowest and highest levels of each factor), permits estimation of the main effects and the two-factor interactions. The axial points allow estimation of the quadratic (curvature) terms and are defined by the coordinates (±α, 0, 0), (0, ±α, 0), and (0, 0, ±α). The centre points, with all factors held at the coded 0 level, provide an estimate of pure experimental error and improve the precision of the quadratic coefficients.

The axial distance was set to α = 1, which places the axial points on the faces of the cubic design space rather than outside it. This corresponds to a face-centred central composite design (FCCD/CCF), in which each factor is studied at only three levels (−1, 0, +1). This configuration was selected so that all parameter settings remained within the operable and physically feasible range of the printer, avoiding the extrapolated extreme settings that a rotatable design (α > 1, five levels) would require.

The 18 experimental runs comprise 8 factorial points (2^3^), 6 axial points (2 × 3), and 4 centre points, i.e., 8 + 6 + 4 = 18. The four replicated centre points supply the degrees of freedom needed to estimate pure error and to perform the lack-of-fit test. All 18 runs were executed in randomized order to minimize the influence of uncontrolled or systematic sources of variation (e.g., machine warm-up and ambient drift). The coded and actual values of the parameters are presented in Table 1, and the experimental layout with the corresponding results is summarized in Table 2.

## 3. Results and Discussion

### 3.1. Regression Modelling

The expression *Strength = F* (*A*, *B*, *C*) describes the relationship between the FDM temperature parameters, namely the post-treatment temperature (A), the nozzle temperature (B), and the bed temperature (C), and the response variables, that is, the tensile strength and the flexural strength. The second-order polynomial regression model, which is used to represent the output response (*y*), can be written as (Equation (1)):

(1) $$y=\beta_{0}+\sum_{i=1}^{k}\beta_{i}X_{i}+\sum_{i=1}^{k}\beta_{ii}X_{i}^{2}+\sum_{i,j=1}^{k.}\beta_{ij}X_{i}X_{j}$$

In this Equation (1), β represents the regression coefficients, which quantify the effect of each input variable on the output. The variable *y* denotes the output response (such as tensile and flexural strength), while *X* represents the input variables (such as nozzle temperature, bed temperature, and post-treatment temperature). The parameter *k* refers to the total number of input variables included in the model. Together, these elements form the basis of the second-order polynomial regression, capturing both the linear and interaction effects of the input parameters on the output response. Further, $\beta_{0}$ is the intercept, $\beta_{i}$ represents the linear coefficients for each input variable, $\beta_{ii}\;$ represents the quadratic coefficients, and $\beta_{ij}$ represents the interaction terms between variables $X_{i}$ and $X_{j}$. The model provides a statistical framework for evaluating the influence of process-parameter variations on the mechanical strength of FDM-printed components. By considering post-treatment temperature, nozzle temperature, and bed temperature as input factors, the model quantifies their individual, quadratic, and interaction effects on tensile and flexural strength. This approach enables the identification of suitable processing conditions for improving the mechanical performance of FDM-printed PEEK parts within the investigated design space.

In the current design analysis, the output variables (tensile and flexural strength) in relation to three factors such as post-treatment temperature (A), nozzle temperature (B), and bed temperature (C) can be represented by a quadratic model as in Equation (2):

(2) $$y=\beta_{0}+\beta_{1}A+\beta_{2}B+\;\beta_{3}C+\;\beta_{11}\;A^{2}\;+\;\beta_{22}B^{2}+\;\beta_{33}C^{2}\;+\;\beta_{12}AB\;+\;\beta_{13}AC\;+\;\beta_{23}BC$$

Using the experimental results, the quadratic model coefficients were calculated through the regression method using the design expert software package. The quadratic equations for tensile and flexural strength were obtained by regression analysis and are presented in Equations (3) and (4), respectively.

*Tensile strength* = 39.93 + 8.56*A +* 6.29*B* − 0.8360*C* − 0.6462*AB* − 1.06*AC* + 1.54*BC* + 3.15*A*^2^ − 6.06*B*^2^ + 0.4367*C*^2^ (3)

*Flexural strength* = 59.20 + 11.30*A* + 8.54*B* − 1.24*C* − 0.83*AB* − 0.70*AC* + 2.16*BC* + 6.76*A*^2^ − 9.90*B*^2^ + 0.75*C*^2^ (4)

The fit summary of the quadratic model is shown in Table 3. For both the tensile and flexural strength, the quadratic model R^2^ values were above 95%, which is close to 1. This confirms that the quadratic model is valid.

**Figure 1:**
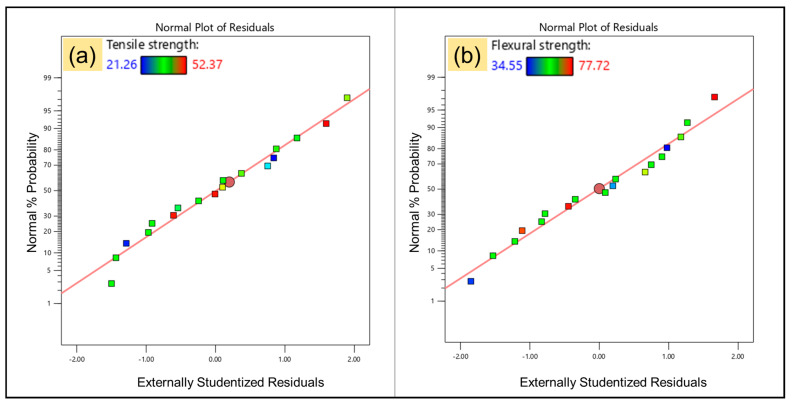
Probability plots for (**a**) tensile strength and (**b**) flexural strength.

**Figure 2:**
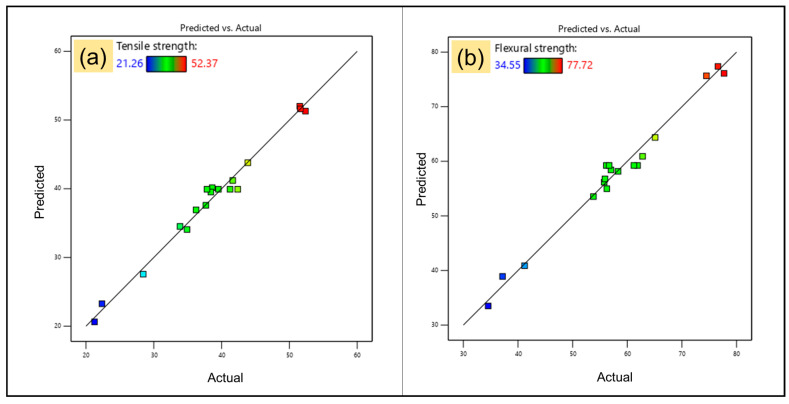
Comparison between experimental results and predicted values for (**a**) tensile strength and (**b**) flexural strength.

The probability plot of the residuals for tensile and flexural strength is illustrated in Figure 1. The data points align closely with the straight line, which indicates that the residuals follow a normal distribution. In contrast, Figure 2 shows the deviation between the actual and predicted values. It is evident that the predicted values closely correspond to the experimental data, reflecting a strong agreement. The predicted and experimental values showed close agreement, indicating that the quadratic models adequately described the response trends within the investigated design space. The subsequent confirmation experiment further supported the model reliability, with prediction errors of 5.63% for tensile strength and 4.08% for flexural strength.

### 3.2. Analysis of Developed Mathematical Models

The regression models and the FDM parameters were evaluated using analysis of variance (ANOVA) to determine their influence on the tensile and flexural strengths. A high model F-value indicates that the model is statistically significant. The ANOVA results for tensile and flexural strength are presented in Table 4 and Table 5, respectively. For tensile strength, the quadratic model was statistically significant, with an F-value of 54.00 and a *p*-value of less than 0.0001, indicating that the model explained a substantial proportion of the observed variation. The post-treatment temperature (A) was the most influential factor, contributing 56.48% of the total variation, with an F-value of 278.98 and a *p*-value of less than 0.0001. The nozzle temperature (B) was the second most significant factor, accounting for 30.56% of the variation, with an F-value of 150.97 and a *p*-value of less than 0.0001. In contrast, the bed temperature (C) exhibited a relatively small contribution of 0.54% and was statistically insignificant (*p* = 0.1413). Among the interaction terms, BC was significant for tensile strength (*p* = 0.0274), indicating that the combined effect of nozzle temperature and bed temperature influenced the tensile response, although the individual effect of bed temperature was not significant.

The tensile model showed a non-significant lack of fit (F = 0.47, *p* = 0.7862), indicating that the quadratic model adequately represented the tensile-strength response within the investigated design space. The model exhibited strong statistical performance, with an R^2^ of 98.38%, an adjusted R^2^ of 96.56%, and a predicted R^2^ of 90.25%, confirming that a large proportion of the response variability was explained by the selected factors. Therefore, the tensile model was considered reliable for identifying the dominant processing parameters and for prediction within the investigated design space. For flexural strength, the quadratic model showed a non-significant lack of fit (*p* = 0.8917), indicating good agreement between the model and the experimental data. Consequently, the flexural strength model was considered more reliable for prediction and optimization within the investigated design space.

For flexural strength, the quadratic model was highly significant, with an F-value of 42.74 and a *p*-value of less than 0.0001, indicating that the model effectively captured the variation in the response. The model explained 97.96% of the total variability, demonstrating excellent agreement between the experimental and predicted values. The post-treatment temperature (A) was the most influential factor, contributing 52.73% of the total variation, followed by nozzle temperature (B), which contributed 30.15%. In contrast, bed temperature (C) had a comparatively small and statistically insignificant individual effect, contributing only 0.64% of the variation.

Among the interaction terms, AB and AC were not statistically significant. However, the BC interaction, corresponding to nozzle temperature × bed temperature, was statistically significant, with an F-value of 6.06 and a *p*-value of 0.0392. This indicates that although bed temperature alone had a limited effect on flexural strength, its combined influence with nozzle temperature was meaningful. The quadratic effects of post-treatment temperature (A^2^) and nozzle temperature (B^2^) were also significant, confirming the presence of curvature in the response surface. Furthermore, the lack-of-fit test was not significant (F = 0.29, *p* = 0.8917), indicating that the residual variation was mainly due to experimental error rather than model inadequacy. Therefore, the quadratic model was considered statistically adequate and reliable for predicting and optimizing flexural strength within the investigated design space.

### 3.3. Parameter Influence on Tensile Strength

**Figure 3:**
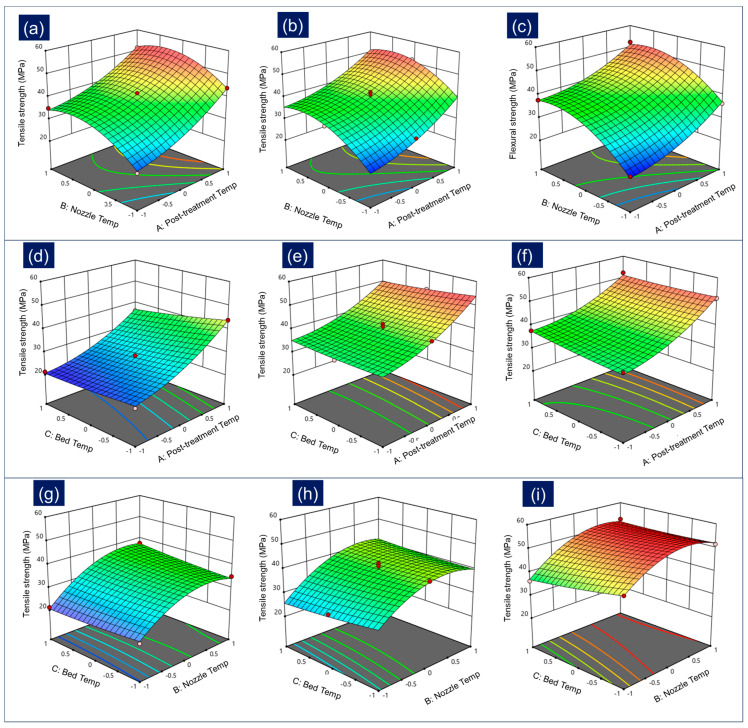
3D surface graph for interaction effects of FDM parameters on tensile strength.

The 3D response surface plots in Figure 3 illustrate the interaction effects of post-treatment temperature, nozzle temperature, and bed temperature on the tensile and flexural strength of the material. Figure 3a–c show the interaction between post-treatment temperature and nozzle temperature, a clear upward trend in tensile strength was observed as both temperatures increased. This improvement in tensile strength was attributed to the enhanced interlayer bonding facilitated by higher nozzle temperatures during extrusion, promoting better adhesion between deposited layers. Additionally, the elevated post-treatment temperature may be associated with polymer-chain relaxation and a possible increase in crystallinity, which would improve the material’s load-bearing capacity and tensile strength [27,28,29]. These synergistic effects were most evident in the red regions of the plots, which represented higher strength values. Figure 3d–f show the interaction between post-treatment and bed temperatures, which indicates the dominant influence of post-treatment temperature in enhancing tensile strength, while bed temperature had a relatively minor effect. The post-treatment process enhanced the material’s structural integrity by relieving internal stresses, which may be associated with more uniform molecular arrangement, whereas the bed temperature mainly influenced the initial layer bonding and overall dimensional stability during the build process [30,31]. Figure 3g–i show the interaction between nozzle and bed temperatures, which highlights the dominant influence of nozzle temperature on tensile strength. As nozzle temperature increased, tensile strength significantly improved, due to better fusion of extruded material layers. Bed temperature had a smaller, yet relevant, effect, particularly when combined with optimized nozzle temperatures, suggesting that controlling bed temperature contributed to minimizing thermal gradients and warping, thereby further enhancing mechanical properties.

To place the present tensile results within the wider FDM-PEEK literature, the optimized confirmation value of 55.65 MPa is consistent with the moderate strengths reported for unfilled PEEK printed under comparatively restricted thermal conditions. Deng et al. [32], using a printing temperature of 370 °C, 40% infill density and no post-treatment annealing, reported an optimized integrated tensile strength of approximately 40 MPa. The higher value obtained in the present study can be attributed to the higher nozzle temperature of 414 °C, the higher infill density of 80%, and the additional annealing step at 200 °C. Substantially higher tensile strengths have been reported when the processing window was extended beyond the range examined in the present work. Li and Lou [33] achieved a tensile strength of 87.34 MPa using a nozzle temperature as high as 525 °C, combined with favourable layer thickness, printing direction and printing path. Similarly, Sikder et al. [34] reported a tensile strength of 97.34 MPa for post-annealed FDM-PEEK specimens, with properties approaching those of injection-moulded PEEK. The comparatively moderate tensile strength obtained in the present study is therefore mainly attributable to the deliberately constrained process window, namely nozzle temperature ≤ 420 °C, bed temperature ≤ 160 °C and annealing temperature ≤ 200 °C. This processing window was selected to preserve the as-printed geometry of the free-standing specimens rather than to maximize crystallinity. Accordingly, the present value should be interpreted as the tensile performance achievable under relatively safe, distortion-limited processing conditions, rather than as the upper mechanical limit of FDM-printed PEEK. A consolidated quantitative comparison across representative FDM-PEEK studies is provided in Section 3.4 and Table 6.

### 3.4. Parameter Influence on Flexural Strength

**Figure 4:**
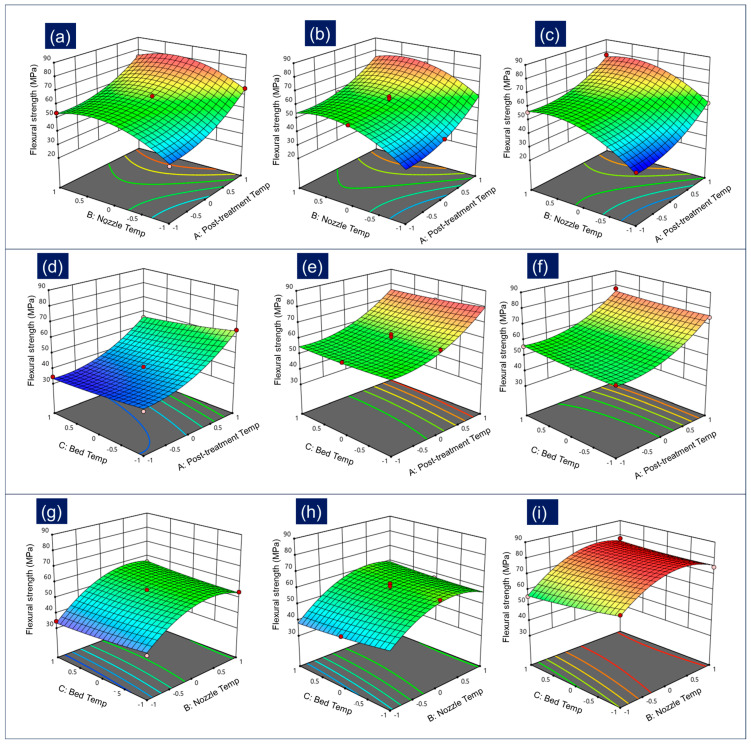
3D surface graph for interaction effects of FDM parameters on flexural strength.

The 3D response surface plots in Figure 4 show the interaction effects of post-treatment temperature, nozzle temperature, and bed temperature on flexural strength. Figure 4a–c demonstrate the interaction between the temperature of post-treatment and nozzle temperature; an increase in flexural strength was found with an increase in both of these parameters. This was greatly contributed to by the fact that layer cohesion and the creation of voids at high nozzle temperatures were minimized hence bonding between the deposited layers was stronger. Also, the temperatures in the post-treatment stage were critical for alleviating internal stresses and may also be associated with molecular reorganization, which could contribute to improved structural integrity and flexural strength [30,31]. The strong curvature of the response surfaces illustrated the synergistic effect between post-treatment and nozzle temperature that reflects the combined effect of the two in improving the flexural performance of the material. Figure 4d–f illustrate the relationship between post-treatment and bed temperature, which indicated that post-treatment temperature had a strong effect on flexural strength. Just as in the case of the tensile properties, bed temperature was a rather minor factor that affected flexural strength. Figure 4g–i reveal the interaction of nozzle temperature and bed temperature, with nozzle temperature being the most important factor that influences flexural strength. The increased temperature of the nozzle facilitated more interlayer fusion resulting in superior mechanical characteristics. The temperature of the bed, though with a less significant impact, assisted in the preservation of the dimensional accuracy, as well as the reduction in warping in combination with the best nozzle settings. However, the effect of bed temperature on flexural strength was still second to the effect of nozzle and post-treatment temperature.

Overall, the tensile strength and flexural strength response surface analysis reveals that the post-treatment temperature and nozzle temperature were the conditions that were essential to the realization of the optimum tensile strength and flexural strength. These results indicate that the better interlayer bonding, stress release and molecular arrangement associated with nozzle and post-treatment temperatures may have contributed to the enhanced mechanical behaviour of the material, with bed temperature being a supplementary factor in the realization of dimensional stability and elimination of residual stresses [30].

**Figure 5:**
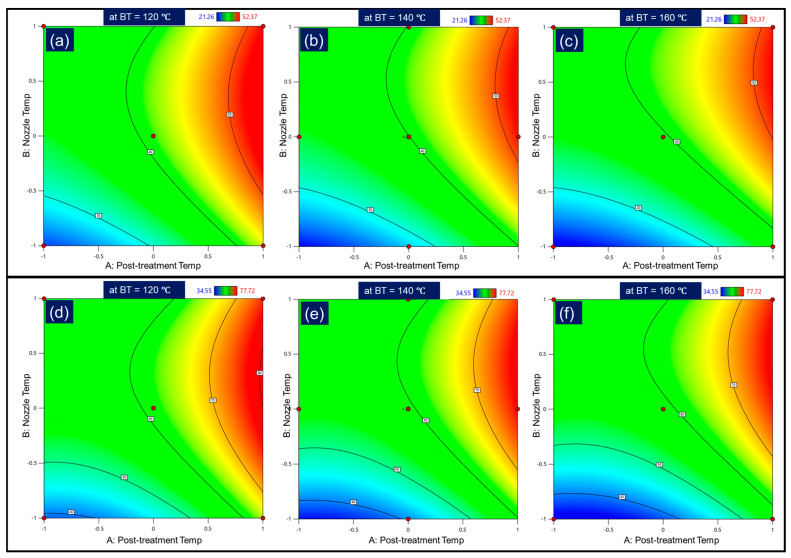
Contour plot for tensile (**a**–**c**) and flexural strength (**d**–**f**). (*BT—bed temperature*).

Figure 5 showed the interaction effect of post-treatment temperature and nozzle temperature on tensile and flexural strength by the use of the contour plots. It is evident that little impact was observed on the effect of bed temperature. It is also realized that the optimization of both the post-treatment temperature and nozzle temperature played an important role in optimizing the tensile and flexural properties of the material, presumably because of the overall synergistic effects of enhanced interlayer bonding, stress alleviation, and inter-directionality between polymer chains during processing [27,28,29]. These findings provide useful guidance for modifying processing conditions to improve the mechanical behaviour of 3D-printed materials in similar applications.

**Figure 6:**
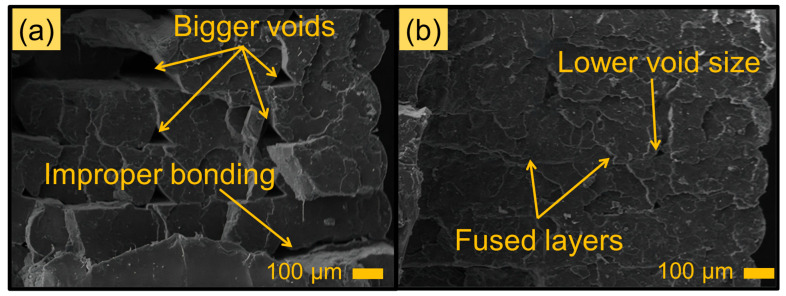
SEM fracture-surface images of tensile specimens showing interlayer bonding and fracture morphology (**a**) before and (**b**) after post-treatment annealing.

The qualitative microstructural evidence for the effect of post-treatment and nozzle temperatures on the microstructure and associated tensile strengths of the material was given by the microstructural analysis of the fractured samples using SEM images. Figure 6 depicts the SEM images of the samples that were fabricated at low nozzle temperature and after treatment at low temperatures expressed the poor interlayer adhesion (experiment number 13), which was manifested by observing the presence of voids and delamination between the layers. Such voids were a source of diminished stress-bearing capacity since the absence of adequate polymer-chain movement and energy during this reduced temperature did not allow efficient fusion of the printed layers, which allowed weak interfaces and reduced tensile strength [27,28,29]. On the other hand, the images of samples processed at higher nozzle and post-treatment temperatures (experiment number 5) had much better interlayer bonding with fewer voids and a more homogeneous structure with well-fused layers. The higher temperatures provided greater mobility for the molecules and may have promoted chain mobility, interlayer diffusion, and possible crystallinity-related structural ordering, resulting in more dense and strong layers [27,28,29]. A reduction in visible voids may be associated with improved performance and density of the material. The difference between the two figures (Figure 6a,b) shows that factors such as temperature, pressure, and material feed rate should be adjusted to make sure that there is sufficient bonding and fewer voids are created. This increased microstructural integrity was observed in SEM images which had smoother and continuous surface morphologies. These microarchitectural enhancements were consistent with the increase in tensile strength observed in the response surface analysis and this validated the importance of thermal processing in enhancing material performance.

**Figure 7:**
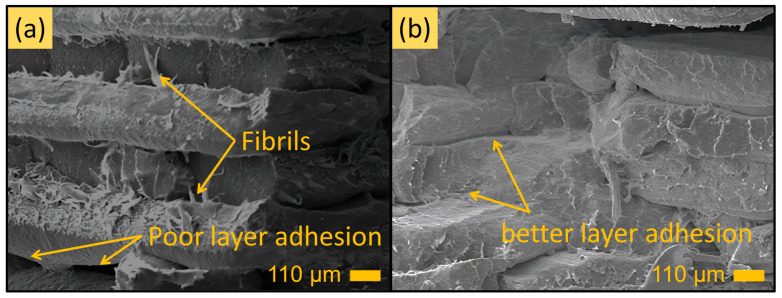
SEM fracture-surface images of flexural specimens showing poor layer adhesion in (**a**) untreated specimens and improved interlayer cohesion after (**b**) post-treatment annealing.

The SEM image of the flexural fracture surface is shown in Figure 7. Figure 7a shows the fibril-like structures on the layers and the poor adhesion between the layers. The distinct layered structure indicates the weak interlaminar bonding between layers. This is evident from areas showing separation between layers, suggesting that inadequate thermal fusion during the printing process contributed to the fracture. The fibrillar structures observed on the fracture surface were the result of inadequate layer adhesion during printing, where the extruded layers did not fully fuse together. This results in uneven, tear-like features along the fracture surface. However, such failures were not found in the samples which underwent the post-treatment process. Figure 7b shows the SEM images of the post-treated samples which show reduced layer separation, with cohesive and tightly bonded layers, indicating stronger interlayer adhesion. This was likely due to enhanced diffusion of polymer chains across layer boundaries during the post-treatment process, reducing weak points where fractures initiate [27,28,29]. The erosion of the fibrillar structures also points to fewer visible void-related defects and reduced interlayer separation in the examined SEM regions, which usually act as stress concentrators. These observations suggest that the post-treatment may be associated with an increase in the crystallinity of the PEEK material, which could increase its strength and stiffness, since crystalline regions are less susceptible to deformation [27,28,29]. In addition, the residual stresses formed during printing are likely to have been reduced by the thermal relaxation provided by the post-treatment, which would further improve the mechanical performance. Together with stronger interlayer bonding, fewer defects, higher ductility, and a possible increase in crystallinity, the post-treatment provided the higher strength measured in the post-annealed FDM-printed PEEK samples [27,28,29]. These observations can be explained by the molecular processes that occur during annealing. During FDM deposition the extruded filaments cool rapidly, which freezes the polymer chains in a non-equilibrium configuration and generates residual stresses across adjacent layers. Post-treatment annealing supplies thermal energy that raises chain mobility, allowing macromolecular segments near the interlayer interfaces to relax, rearrange, and diffuse across the layer boundaries; this improves interlayer cohesion, reduces stress-concentration sites, and promotes further ordering of the semi-crystalline PEEK structure, although the degree of crystallinity was not directly quantified in this study. These mechanisms account for the improved tensile and flexural strength measured after post-treatment. Comparable behaviour, in which heat treatment and thermal history govern crystallinity, interlayer diffusion, residual-stress relaxation, and porosity reduction, has been reported for other additively manufactured semi-crystalline polymers and polymer composites [27,28,29,35].

To benchmark the mechanical performance achieved in the present study against comparable work, Table 6 summarizes the key thermal processing conditions, particularly nozzle and annealing temperatures, together with the corresponding tensile and flexural strengths reported for representative FDM-printed PEEK studies. The reported values span a wide range, reflecting the strong sensitivity of FDM-printed PEEK to thermal history, infill density, layer bonding, build orientation, and post-processing conditions.

Several factors account for the spread of values in Table 6. First, the nozzle and annealing temperatures differ markedly between studies. The highest strengths were obtained either at very high nozzle temperatures, such as 525 °C in the work of Li and Lou [33], or after higher-temperature annealing close to the crystallization region, as reported by He et al. [10]. These thermal conditions promote greater crystallinity, improved interlayer diffusion, and stronger filament-to-filament bonding than the constrained processing window adopted in the present study. He et al. [10], for example, reported as-printed tensile strengths in the range of 60–70 MPa, which increased by approximately 36% in tension and 54% in flexure after annealing at 300 °C. The controlling role of thermal history in determining crystallinity and, consequently, the strength of printed PEEK has also been demonstrated previously [31].

Second, FDM-printed PEEK is intrinsically anisotropic; therefore, reported strengths depend strongly on build orientation, raster angle, printing path, and loading direction. Horizontally printed or load-aligned specimens generally exhibit higher tensile performance than vertically printed specimens because the applied load is carried more effectively along the deposited roads rather than across weak interlayer interfaces. Third, the persistence of inter-bead voids, incomplete fusion, and weak layer interfaces reduces the effective load-bearing cross-section and introduces stress-concentration sites. These defects are governed by infill density, layer height, nozzle temperature, chamber temperature, print speed, and bonding quality. Consequently, even optimized FDM-printed PEEK parts commonly remain below fully consolidated injection-moulded PEEK, which typically exhibits tensile strengths of approximately 90–105 MPa and flexural strengths of approximately 125–165 MPa, depending on grade and testing condition.

Finally, differences in feedstock grade, printer configuration, specimen geometry, and test standard, such as ASTM D638/ASTM D790, ISO 527(ISO 527-1:2019; Plastics—Determination of Tensile Properties—Part 1: General Principles. International Organi-zation for Standardization: Geneva, Switzerland, 2019.)/ISO 178 (ISO 178:2019; Plastics—Determination of Flexural Properties. International Organization for Standardization: Ge-neva, Switzerland, 2019.), or GB/T methods (GB/T 1040.1-2025; Plastics—Determination of Tensile Properties—Part 1: General Principles. State Administration for Market Regulation and Standardization Administration of China: Beijing, China, 2025.), further contribute to scatter among reported values. In the present work, the thermal window was deliberately constrained to preserve specimen geometry, with nozzle temperature limited to ≤420 °C, bed temperature to ≤160 °C, and annealing temperature to ≤200 °C. Therefore, the moderate tensile and flexural strengths reported here should be interpreted as the performance attainable under relatively safe, distortion-limited processing conditions rather than as the upper bound of FDM-printed PEEK. Extending the nozzle or annealing temperature, as reported in [33,34], would be expected to narrow the remaining gap relative to injection-moulded PEEK, but at the cost of an increased risk of warpage, dimensional distortion, or loss of geometric fidelity in free-standing printed specimens.

### 3.5. Numerical Optimization and Confirmation Experiment

**Figure 8:**
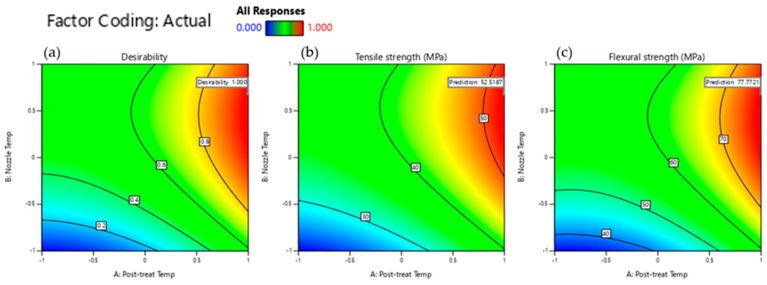
Desirability-based numerical optimization of FDM-printed PEEK using RSM. The contour plots show (**a**) overall desirability, (**b**) tensile strength, and (**c**) flexural strength.

Numerical optimization was carried out using the desirability function approach in Design-Expert. The three input factors, namely post-treatment temperature, nozzle temperature, and bed temperature, were maintained within the investigated experimental range, while tensile strength and flexural strength were both set to be maximized. The experimental range used in the RSM design was 100–200 °C for post-treatment temperature, 380–420 °C for nozzle temperature, and 120–160 °C for bed temperature. The optimized solution predicted by the model was obtained at a coded post-treatment temperature of 0.995, nozzle temperature of 0.678, and bed temperature of 0.097. These coded values correspond to approximately 199.8 °C post-treatment temperature, 413.6 °C nozzle temperature, and 141.9 °C bed temperature. Under this optimum condition, the model predicted a tensile strength of 52.519 MPa and a flexural strength of 77.772 MPa, with an overall desirability value of 1.000. The desirability and response contour plots shown in Figure 8 confirm that the optimum region is located near the upper post-treatment temperature range and moderately high nozzle temperature, where both tensile and flexural responses are simultaneously maximized. For practical implementation, the predicted parameter values were rounded to the nearest feasible machine settings, namely 200 °C post-treatment temperature, 414 °C nozzle temperature, and 142 °C bed temperature. A confirmation experiment was then conducted using these rounded optimized parameters. The optimized parameter set, predicted responses, experimental confirmation results, and corresponding prediction errors are summarized in Table 7. The experimentally measured tensile strength and flexural strength were 55.65 MPa and 81.08 MPa, respectively. The corresponding prediction errors were 5.63% for tensile strength and 4.08% for flexural strength. These low error values confirm that the developed RSM models can reliably predict the mechanical performance of FDM-printed PEEK within the investigated design space. It is useful to place these values in the context of conventionally processed PEEK. The optimized FDM-printed specimens reached a confirmation tensile strength of 55.65 MPa and a flexural strength of 81.08 MPa, which remain below the values typically reported for injection-moulded or otherwise fully consolidated unfilled PEEK (tensile strength of ca. 95–116 MPa and flexural strength of ca. 125–175 MPa, depending on grade and test standard). This difference is expected because FDM parts contain layer interfaces, thermal gradients, and possible inter-bead voids that reduce mechanical performance relative to fully dense moulded PEEK. Nevertheless, the present results show that thermal-parameter optimization combined with post-treatment annealing can substantially improve FDM-printed PEEK, bringing it to a level that may be suitable for non-critical or moderately loaded functional components, customized biomedical parts, fixtures, and thermally resistant polymer components. Further validation under fatigue, creep, impact, and long-term service conditions would be required before deployment in critical aerospace, automotive, or load-bearing biomedical applications. From an industrial standpoint, the optimized route requires a high-temperature-capable FDM system (nozzle ~414 °C with a heated bed and an enclosed, actively heated chamber) and an additional one-hour annealing step, which raise energy consumption, equipment cost, and total cycle time relative to commodity-polymer FDM. Annealing is nevertheless a simple and scalable batch operation, since many parts can be processed in a single oven cycle, and high-temperature FDM hardware is increasingly available commercially; the approach is therefore economically reasonable for high-value, customized, or biomedical PEEK components where the material’s properties are required, while for low-value or high-throughput parts the additional cost may not be justified. The main limitations are reduced throughput due to the annealing time, higher energy and equipment cost, and the need for careful fixturing to prevent distortion during annealing; a dedicated techno-economic optimization is a useful direction for future work.

## 4. Conclusions

This study investigated the influence of post-treatment annealing temperature, nozzle temperature, and bed temperature on the tensile and flexural strength of FDM-printed PEEK using Response Surface Methodology. A face-centred central composite design was successfully applied to model the effects of the selected thermal parameters and to identify an optimized processing condition for improving mechanical performance. The results showed that post-treatment temperature was the most influential parameter, contributing 56.48% to tensile strength and 52.73% to flexural strength. Nozzle temperature was the second most important factor, contributing 30.56% and 30.15% to tensile and flexural strength, respectively, whereas bed temperature had only a minor individual effect within the selected range. However, the nozzle temperature–bed temperature interaction significantly affected both tensile and flexural strength, indicating that bed temperature can still influence mechanical performance when combined with an appropriate nozzle temperature. The optimized condition predicted by the desirability approach was approximately 199.8 °C post-treatment temperature, 413.6 °C nozzle temperature, and 141.9 °C bed temperature. The confirmation experiment, conducted at 200 °C, 414 °C, and 142 °C, produced a tensile strength of 55.65 MPa and a flexural strength of 81.08 MPa, with prediction errors of 5.63% and 4.08%, respectively. These results confirm the reliability of the developed models within the investigated design space. SEM observations qualitatively indicated improved interlayer bonding, fewer visible void-related defects, and a more cohesive fracture morphology after optimized thermal processing. These improvements are likely related to enhanced interlayer fusion, increased polymer-chain mobility, and thermal-stress relaxation during post-treatment annealing.

## Figures and Tables

**Table 1:** Experimental parameters and their coding.

Coded Level	Post-Treatment Temperature (°C)	Nozzle Temperature (°C)	Bed Temperature (°C)
−1	100	380	120
0	150	400	140
+1	200	420	160

**Table 2:** Experiment design and results.

S. No.	Input Variables			Output Variables	
	Post-Treatment Temperature (Coded)	Nozzle Temperature (Coded)	Bed Temperature (Coded)	Tensile Strength (MPa)	Flexural Strength (MPa)
1	−1	0	0	33.87	55.26
2	−1	−1	1	21.26	34.55
3	0	−1	0	28.45	41.22
4	1	0	0	51.62	76.6
5	1	1	1	52.37	77.72
6	−1	1	1	37.68	55.92
7	0	0	−1	41.65	62.81
8	0	0	1	38.42	57.02
9	−1	1	−1	34.91	53.81
10	1	−1	1	36.24	55.78
11	0	0	0	37.85	56.17
12	1	1	−1	51.54	74.49
13	−1	−1	−1	22.36	37.18
14	1	−1	−1	43.87	65.1
15	0	0	0	39.54	56.68
16	0	0	0	41.25	61.22
17	0	1	0	38.62	57.31
18	0	0	0	42.38	62.89

**Table 3:** Fitness of the quadratic model.

Test	Source	R^2^	Adjusted R^2^	Predicted R^2^	Remarks
Tensile Strength	Quadratic	98.38	96.56	90.25	Suggested
Flexural Strength	Quadratic	97.96	95.67	87.86	Suggested

**Table 4:** ANOVA for quadratic model: Tensile strength.

Source	Sum of Squares	df	Mean Square	F-Value	*p*-Value	Contribution %
**Model**	1275.24	9	141.69	54.00	<0.0001	98.38
A—Post-Treat Temp	732.05	1	732.05	278.98	<0.0001	56.48
B—Nozzle Temp	396.14	1	396.14	150.97	<0.0001	30.56
C—Bed Temp	6.99	1	6.99	2.66	0.1413	0.54
AB	3.34	1	3.34	1.27	0.2919	0.26
AC	8.97	1	8.97	3.42	0.1017	0.69
BC	19.00	1	19.00	7.24	0.0274	1.47
A^2^	26.83	1	26.83	10.22	0.0127	2.07
B^2^	99.62	1	99.62	37.96	0.0003	7.69
C^2^	0.52	1	0.52	0.20	0.6690	0.04
Residual	20.99	8	2.62			1.62
Lack of Fit	9.19	5	1.84	0.47	0.7862	0.71
Pure Error	11.80	3	3.93			0.91
Cor Total	1296.24	17				

**Table 5:** ANOVA for quadratic model: Flexural strength.

Source	Sum of Squares	df	Mean Square	F-Value	*p*-Value	Contribution %
**Model**	2370.78	9	263.42	42.74	<0.0001	97.96
*A—Post-Treat Temp*	1276.22	1	1276.22	207.06	<0.0001	52.73
*B—Nozzle Temp*	729.66	1	729.66	118.39	<0.0001	30.15
*C—Bed Temp*	15.38	1	15.38	2.49	0.1529	0.64
*AB*	5.56	1	5.56	0.90	0.3700	0.23
*AC*	3.88	1	3.88	0.63	0.4505	0.16
*BC*	37.37	1	37.37	6.06	0.0392	1.54
*A* ^2^	123.90	1	123.90	20.10	0.0020	5.12
*B* ^2^	265.74	1	265.74	43.12	0.0002	10.98
*C* ^2^	1.51	1	1.51	0.25	0.6337	0.06
Residual	49.31	8	6.16			2.04
Lack of Fit	16.09	5	3.22	0.29	0.8917	0.66
Pure Error	33.22	3	11.07			1.37
Cor Total	2420.09	17				

**Table 6:** Comparison of the present optimized FDM-PEEK results with representative FDM-printed PEEK studies. TS = Tensile strength; FS = flexural strength.

Study (Ref.)	Nozzle Temp. (°C)	Annealing Temp. (°C)	TS (MPa)	FS (MPa)
Deng et al. [32]	370	— (as-printed)	40.0	68.2
He et al. [10]	440	up to 300	60–70 *as-printed*	*—*
Li and Lou [33]	up to 525	— (as-printed)	87.34	159.2
Sikder et al. [34]	410	160 + 200	97.34	104.65
Present study	414	200	55.65	81.08

**Table 7:** RSM optimization confirmation results.

Setting	TS, MPa	FS, MPa	Error, %	Desirability
Predicted optimum: 199.8 °C/413.6 °C/141.9 °C	52.52	77.77	—	1.000
Confirmation experiment: 200 °C/414 °C/142 °C	55.65	81.08	5.63/4.08	—

## Data Availability

The original contributions presented in this study are included in the article. Further inquiries can be directed to the corresponding author.
